# Assessment of Methodological Pipelines for the Determination of Isothiocyanates Derived from Natural Sources

**DOI:** 10.3390/antiox11040642

**Published:** 2022-03-27

**Authors:** Sotiris Kyriakou, Dimitrios T. Trafalis, Maria V. Deligiorgi, Rodrigo Franco, Aglaia Pappa, Mihalis I. Panayiotidis

**Affiliations:** 1 Department of Cancer Genetics, Therapeutics & Ultrastructural Pathology, The Cyprus Institute of Neurology & Genetics, Ayios Dometios, Nicosia 2371, Cyprus; sotirisk@cing.ac.cy; 2 Laboratory of Pharmacology, Medical School, National & Kapodistrian University of Athens, 11527 Athens, Greece; dtrafal@med.uoa.gr (D.T.T.); mdeligiorgi@yahoo.com (M.V.D.); 3 Redox Biology Centre, University of Nebraska-Lincoln, Lincoln, NE 68583, USA; rodrigo.franco@unl.edu; 4 Department of Veterinary Medicine & Biomedical Sciences, University of Nebraska-Lincoln, Lincoln, NE 68583, USA; 5 Department of Molecular Biology & Genetics, Democritus University of Thrace, 68100 Alexandroupolis, Greece; apappa@mbg.duth.gr

**Keywords:** isothiocyanates, myrosinase, extraction, glucosinolates, hydrolysis, quantification

## Abstract

Isothiocyanates are biologically active secondary metabolites liberated via enzymatic hydrolysis of their sulfur enriched precursors, glucosinolates, upon tissue plant disruption. The importance of this class of compounds lies in their capacity to induce anti-cancer, anti-microbial, anti-inflammatory, neuroprotective, and other bioactive properties. As such, their isolation from natural sources is of utmost importance. In this review article, an extensive examination of the various parameters (hydrolysis, extraction, and quantification) affecting the isolation of isothiocyanates from naturally-derived sources is presented. Overall, the effective isolation/extraction and quantification of isothiocyanate is strongly associated with their chemical and physicochemical properties, such as polarity-solubility as well as thermal and acidic stability. Furthermore, the successful activation of myrosinase appears to be a major factor affecting the conversion of glucosinolates into active isothiocyanates.

## 1. Introduction

Naturally occurring plant-derived chemical compounds, known as phytochemicals, have been the subject of intense research due to their important role in health promotion and disease prevention [1,2,3,4,5,6,7,8,9,10]. To these ends, numerous epidemiological studies have documented a low consumption of fruits and vegetables conception with a dramatic increase in the rate of cancer development and progression, among other diseases (e.g., cardiovascular disease, metabolic syndrome, etc.) [11,12,13,14,15,16,17,18,19,20,21]. Finally, the role of diet in the prevention of neurodegenerative diseases (e.g., Dementia, Alzheimer’s, Parkinson’s, etc.) has been extensively reviewed by emphasizing on the role of various phytochemicals (e.g., omega-3 fatty acids, flavonoids, vitamins, etc.) by exerting a plethora of disease-preventing biological properties [22,23,24,25].

**Figure 1:**
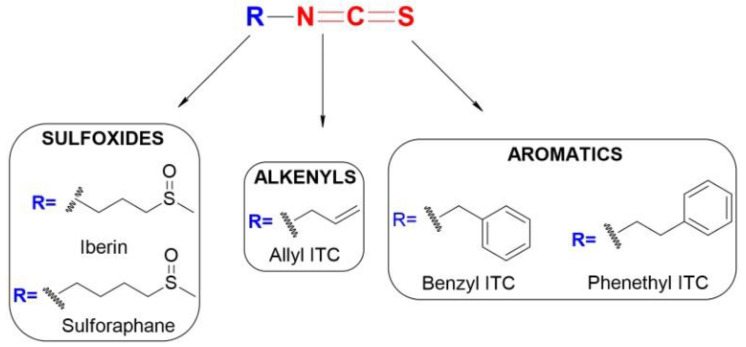
Schematic representation of the segregation of ITCs based on the type of the R group (sulfoxides, alkenyls, and aromatics).

Isothiocyanates (ITCs) are a class of phytochemicals with exceptional biological and nutritional activity. These are aliphatic or aromatic phytochemicals produced in high abundance in cruciferous plants, including broccoli, kale, cauliflower, cabbage, Brussel sprouts, wasabi roots, and watercress. They all have the same chemical backbone, R-N=C=S (where R- can be either an aliphatic or aromatic group; Figure 1), whereas their content varies across species, variety, and growing conditions (Table 1) [26,27].

**Figure 2:**
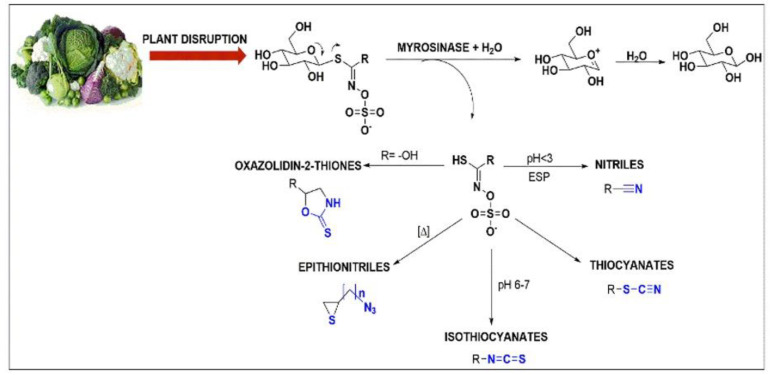
Hydrolysis of GSLs via enzymatic catalysis by myrosinase is activated upon tissue plant disruption. The formed aglucone can be rearranged into oxazolidin-2-thiones, nitriles, epithionitres, thiocyanates, and ITCs.

So far, more than 154 different glucosinolates (GSLs) have been identified and these can be categorised into 4 classes: aliphatic, aromatic, indolyl, and glycosylated [40,41,42,43,44]. The breakdown product of the hydrolysis of GSLs includes a variety of nitrogenous compounds such as ITCs, thiocyanates, nitriles, and epithionitriles, depending on the reaction conditions (i.e., pH, temperature and reducing agents like ferrous ions, ascorbate, etc.) [45,46,47,48,49,50,51,52,53,54]. For instance, thiohydroximate-*O*-sulphate, at neutral pH, is rearranged into ITCs whereas at low pH it can be rearranged into nitriles (Figure 2) [55,56,57,58,59,60,61]. Finally, the presence of epithiospecifier protein-like (ESP) factors can drive the reaction towards the formation of nitriles as well. In addition, ESPs have the capacity to convert alkenyl-contained intermediates into epithionitriles which are considerably less stable products [59,61,62,63]. In all cases, the rearrangement of thiohydroximate-*O*-sulphate into its active metabolites is accompanied by loss of a sulphate group.

**Figure 3:**
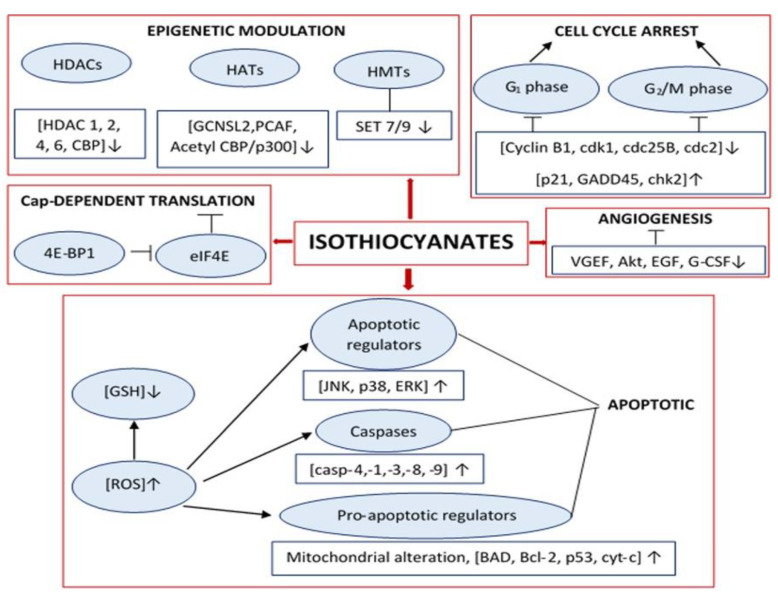
The involvement of ITCs in various cell signalling cascades associated with cell cycle arrest, epigenetic modulation, generation of oxidative stress, angiogenesis, and apoptosis. Arrowheads pointing upwards or downwards indicate an upregulation or downregulation of the expression of various indicated protein markers and ROS, respectively.

The importance of this class of molecules relies on their anticancer properties by downgrading the factors that are responsible for the cellular detoxification [50,64]. More specifically, ITCs can form an adduct with glutathione (GSH) during the mercapturic acid pathway, causing GSH depletion [65,66,67]. As a result, the levels of reactive oxygen species (ROS) are elevated, leading to down-stream activation of caspases and other apoptotic markers, thereby promoting the induction of apoptosis [68,69,70,71]. It has been postulated, throughout the literature, that ITCs can also induce cell cycle growth arrest by down- and/or up-regulating various cycle-dependent kinases (CDKs) and tumour suppressor genes, respectively [72,73,74]. Moreover, their anti-angiogenic capacity has also been suggested, primarily through their ability to down-regulate both vascular endothelial as well as epithelial growth factors [75,76,77]. Finally, they have been shown to affect the epigenome through their capacity to regulate the expression levels of histone deacetylases, acetyltransferases, and methyltransferases, among other epigenetic events (Figure 3) [77,78,79]. In another study, nanoformulation of ITCs facilitates their solubility and stability, leading to an improved bioavailability and anticancer capacity [80]. The nanoformulation of ITCs was acomplished via their encapsulation with either organic (micelle, liposome, dendrimer, polymeric nanoparticles, solid lipoid nanoparticle, and carbon nantube) or inorganic (gold, iron oxide or silicon) nanocarriers [80].

In conclusion, this review article aims to provide an integrated optimum methodological approach towards the isolation and quantification of different classes of ITCs. To this end, the proposed experimental pipeline provides fast and easy access to the isolation and quantification of major ITCs (SFN, IBN, AITC, BITC, and PEITC) from naturally-derived sources.

## 2. Determination of ITC Content

**Figure 4:**
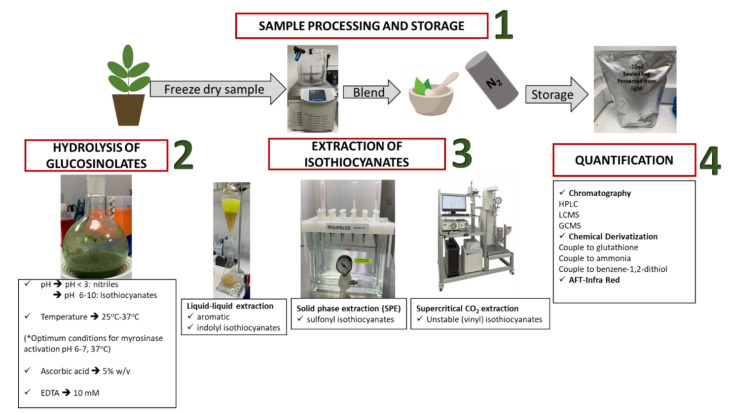
The 4 pillars: (1) sample processing and storage, (2) hydrolysis of GSLs, (3) extraction of ITCs, and (4) quantification for the determination of ITCs from naturally—derived sources.

A general protocol that includes the experimental pipeline for the determination of ITCs content from naturally-derived sources is divided in four pillars, namely (i) sample storage and isolation, (ii) hydrolysis of GSLs, (iii) extraction of ITCs, and eventually (iv) their quantification (Figure 4).

### 2.1. Sample Processing and Storage

Sample preparation prior to analyte identification and quantification, using analytical techniques, is a very crucial step in preventing against analyte loss and/or its decomposition [81,82]. More specifically, cellular decomposition of cruciferous plants (by chopping, boiling, etc.) leads to activation of myrosinase and hence the hydrolysis of GSLs. Therefore, it is recommended that small cruciferous plants or parts of the plant are freeze-dried (at −20 °C) and stored under solid carbon dioxide conditions (−80 °C), whereas larger plants should be immediately frozen under liquid nitrogen to avoid the activation of myrosinase [81,82,83,84].

### 2.2. Hydrolysis of GSLs

#### 2.2.1. Effect of pH

Hydrolysis of GSLs comprises a major role in determining the content of ITCs in cruciferous vegetables (Table 2). 

This can be attributed to the fact that different parameters (such as temperature and pH) can affect the hydrolysis of GSLs, leading to their partial or complete conversion into ITCs and/or other nitrogenous-contained metabolites [109]. In previous studies, it has been demonstrated that different pH environments can affect the (i) formation of different intermediates (e.g., ITCs, nitriles), (ii) purity, and (iii) stability of the final product [60,110]. For example, Vaughn S.F and Berhow M.A. (2005) have shown that hydrolysis of the seeds from the plants *Eruca sativa* var. *sativa* (Aragula) and *Erusimum allionii* (Siberian wallflower) (both being sources of erucin) leads to formation of different products/mixtures (regardless of both plants containing the same GSL precursor), under a range of pH values [85]. Specifically, upon exposure of *Eruca sativa* var. *sativa* to phosphate buffer (at pH 7.0), a mixture of erucin and erucin nitrile was formed, while the GSL precursor of *Erusimum allionii*, under the same conditions, formed erucin, cheirolin, eysolin, and sulforaphane (SFN) [85]. On the other hand, at basic conditions (pH 10.0) both plants produced erucin, while at acidic conditions (pH 1.0), *Eruca sativa* var. *sativa*’s GSL content was completely converted into erucin nitrile [85] and *Erusimum allionii’s* into a mixture of erucin nitrile, sulforaphane nitrile, and erysolin nitrile [86,110]. Moreover, during the same study, exposure of *Brassica hirta* (L.) Moench (white mustard), *Lesquerella fendleri* (Lesquerella), and *Mattiola longipetala* (night-scented stock) at pH 7.0 and 10.0 led to complete hydrolysis of their respective GSLs into 4-hydroxybenzyl ITC, iberin (IBN), and SFN, respectively [85]. Similarly, exposure of *Capsella bursa-pastoris* (L.) Medik (Shepher’s purse), *Lobularia maritima* (L.) Desv. (Sweet alyssum), and *Erysimum cheiri* (L.) Crantz (English wallflower) at pH 7.0 and 10.0, 3-butenyl ITC, cheroline, and lesquerellin were produced as the maim hydrolysis products, whereas none of the GSL precursors were hydrolysed at pH 1.0 [85].

In another study, when hydrolysis of gluconasturtiin (obtained from watercress), under a range of pH values (e.g., 5.0, 7.0, and 9.0) occurred, it was shown that the optimum pH for its conversion into phenethyl ITC (PEITC) was at 7.0 in phosphate buffer [87,88]. In similar studies, hydrolysis of glucoraphanin (derived from *Brassica oleracea* var. *italica*; broccoli) using a citrate/phosphate buffer (at pH 6.5) led to complete conversion into SFN [89]. Furthermore, in studies conducted by other groups, different species of *Brassica oleracea* were utilised (as a source of glucoraphanin) in order to examine the effect of different pH values (e.g., 5.0, 5.5, 6.0, 6.5, 7.0, 7.5, and 8.0) into the bio-production of SFN [88,89,90]. The outcome of these studies demonstrated that although hydrolysis of glucoraphanin was achieved, under all pH values, the content of SFN varied as it was pH-dependent [88,90,91]. Finally, the authors have concluded that pH 7.0 is the optimum condition for myrosinase activity during hydrolysis of sinigrin from *Armoracia rusticana* (horseradish), a finding consistent with the relevant bibliography [92,93].

Finally, in another study, hydrolysis of glucotropaeolin (obtained either from *Carica* papaya (L.) (papaya) or *Tropaeolum majus* (L.) *tropaeolaceae* (garden nasturtium) or *Lepidium sativum* (L.) (garden cress)) yielded benzyl ITC (BEITC), at pH 6.5–7.0, whereas at pH 1.0 there was formation of benzyl nitrile with traces of BEITC [94,95,96,97]. Coscueta et al. (2020) observed that production of PEITC occurred at almost quantitative levels when *Nasturtium officinale* (watercress) was exposed at pH 7.0. At acidic (pH 5.0) or basic (pH 10.0) conditions, the activity of myrosinase was decreased, leading to the formation of either a mixture of PEITC/phenethyl nitrile or phenethyl nitrile, respectively [98]. These findings are in agreement with the studies by Hancshen et al. (2017) and Hanschen and Schreiner (2017), documenting that the hydrolysis of ITCs is enhanced upon exposure to neutral or slightly basic conditions [56,58], as under these experimental conditions the kinetic properties of myrosinase are accelerated, thus driving the reaction towards the formation of ITCs [42,99,100]. On the contrary, under acidic conditions, the reaction’s kinetics can change as this either reduces the activity of myrosinase (i.e., the hydrolysis rate is decreased) or drives the reaction towards complete conversion of GSLs into their respective nitriles. Interestingly, the majority of studies have shown that in slightly acidic pH (1.0 < pH < 7.0), GSL hydrolysis leads to the formation of a mixture of both ITCs and nitriles [61,101].

#### 2.2.2. Effect of Temperature

Another factor of great importance, for the conversion of GSLs into ITCs, is the temperature of the hydrolysis reaction, as it can affect the activity of myrosinase and/or the stability of ITCs [111]. Earlier studies have suggested that myrosinase, extracted from broccoli, is stable at temperatures below 40 °C, whereas at 50 °C the activity decreased by 70% within 5 min of exposure [112]. In contrast, another study has identified high levels of SFN extracts in broccoli after treating the vegetable at 60 °C for 10 min, thereby demonstrating that the myrosinase can be remained active even at 60 °C [113]. In any case, various studies have aimed to characterize the relationship between myrosinase activity and temperature towards conversation of GSLs into ITCs in the *Brassica* family [114,115,116].

On this note, the heat stability of white (*Brassica oleracea* var *capitate*) and red (*Brassica oleracea* var *capitata f. rubra*) cabbages was examined at 70 °C for 30 min, demonstrating reduced production of AITC due to thermal degradation of myrosinase [117,118]. In addition, other studies have shown that 30 °C and 50 °C were optimum temperatures for myrosinase activity in broccoli and Brussels sprouts, respectively [111,119]. Furthermore, hydrolysis of 2-propyl and 3-butyl GSLs into their respective ITCs, in *Arabidopsis thaliana* (L.) Heynth, was higher at 37 °C when compared to other temperature ranges [120]. In another study, the authors examined the stability of the respective enzymes obtained from yellow, brown, and black mustard seeds, showing that their stability tolerance was limited up to 60 °C (in all three species) and that the activity of myrosinase was significantly decreased above 60 °C [112]. In particular, the myrosinase activity from yellow mustard was only stable up to 50 °C, while it lost about 79% of its activity at 60 °C. Moreover, the myrosinase activity in black and brown mustards, at 70 °C, was significantly reduced to 41% and 65%, respectively, whereas in yellow mustard it was completely abolished [112]. The fact that some enzymatic activity has been recorded at temperatures above 85 °C (as small traces of glucosinolate degradation products) could be attributed to the presence of thermostable desulphatase enzymes, which are activated at high temperatures and can catalyse the hydrolysis of thioglucosidic bonds [112]. Finally, these results were further supported by another study indicating that the inactivation of myrosinase in mustard species can occur in just 10 min of exposure at 60 °C [121,122] (Table 3).

Although temperature is a major factor that can affect the formation of ITCs from the hydrolysis of GSLs, it is less stringent than pH. It is suggested that this might be attributed to the different optimum temperatures of the different ESPs of various plants [122,123]. To this end, the presence of ESPs in *Arabidopsis thaliana* (L.) Heynh increases nitrile formation (at lower temperatures (e.g., 0–20 °C)), while myrosinase activity is reduced [123,124,125]. These findings are in contradiction with the results of other studies, indicating that, in *Arabidopsis thaliana* (L.) Heynh, a temperature of 37 °C possesses an inverse effect where the production of ITCs and nitriles is at higher rates. In addition, at 37 °C, the epithionitrile levels are reduced, implying loss of ESP activity [61,126].

In conclusion, myrosinase is a temperature sensitive enzyme and so thermal treatments (including sample heat processing and blanching) can cause a decrease in its activity and consequently in the production of ITCs. In addition, increased formation of ITCs is achieved if ESP activity is not affected and the ratio of glucosinolate-aglucon to ESP increases, thereby leading to lower EPT and higher ITCs formation [127].

#### 2.2.3. Effect of EDTA and Ascorbic Acid

In addition to pH and temperature, the expression levels of ESPs and the presence of Fe^2+^ are secondary factors which can also affect the hydrolysis of GSLs and the production of ITCs. Previous studies have shown that acidic conditions in combination with high levels of ESPs and ferrous ions (Fe^2+^) can lead to hydrolysis reactions into yielding high levels of nitriles and thus undesired toxicity. In addition, activation of ESPs requires Fe^2+^, therefore decreasing the content of ferrous ion which could suppress the formation of nitriles and thus facilitate the production of ITCs. Various studies have suggested that the addition of ascorbic acid can stimulate the action of myrosinase by facilitating the hydrolysis of GSLs [128,129,130,131]. To this end, various attempts have been made in identifying the role of ferrous ions and ascorbic acid on myrosinase activity [132]. On another note, the addition of 10 mM of ethylenediaminetetraacetic acid (EDTA) (at pH 6.8) can lead to a high ITC conversion rate due to its chelating capability in binding and coordinating metal ions (magnesium; Mg^2+^, ferrous; Fe^2+^, ferric; Fe^3+^, etc.), thereby diminishing the presence of metal ions in enzymolytic solutions [128]. In addition, the formation of nitriles was shown to be reduced by decreasing the content of Fe^2+^, in Brussel sprouts, thus enhancing the production of ITCs [133,134]. Overall, the optimum EDTA concentration needed for complete inhibition of ESPs (hence maximal production of ITCs) is of utmost importance in modulating myrosinase activity [133,134].

On the other hand, ascorbic acid has been previously reported to function as a cofactor of myrosinase, thus its availability and concentration can affect its activity [135,136]. Specifically, excessive levels of ascorbic acid can inhibit myrosinase activity (thus less ITCs formation), whereas reduced levels might not lead to optimum levels of myrosinase activity [137]. To this end, it was suggested that the optimum concentration of ascorbic acid, for maximum myrosinase activity, was about 5% *w/v*. This can be attributed to ascorbic acid acting as an uncompetitive activator of myrosinase, although the exact mechanism of such activation is not entirely elucidated [136,137].

## 3. Extraction of Isothiocyanates

Due to their major biological properties, the effective extraction and isolation of ITCs is of utmost importance. This is because an optimum extraction method will allow the maximum recovery and thus a valid determination of ITCs content in cruciferous plants. However, a common extraction procedure for all ITCs cannot be achieved since each ITC has different physical properties, including polarity, volatility, and stability. For this purpose, different procedures have been applied, including liquid–liquid, solid phase, supercritical gas assisted, and ultrasonic extractions. In this section the extraction of the most utilized and abundant ITCs will be discussed, including SFN, IBN, AITC, BITC, and PEITC.

### 3.1. Extraction of Sulforaphane

The enzymatic hydrolysis of glucoraphane generates a variety of compounds (e.g., glucose, sulphates, ITCs, thiocyanates, nitriles, etc.) which interfere with the separation and determination of SFN [138,139,140]. Therefore, it is necessary to establish a simple and convenient method for the selective extraction and separation of SFN from various cruciferous vegetable sources, including broccoli. In most studies, the isolation of SFN is accompanied with the hydrolysis of its precursor GSL (i.e., glucoraphane) by treating the plant (or parts of the plant) with water, at pH 6.0–7.0, under various temperatures (e.g., 25–37 °C), followed by a further extraction step with a solvent of medium polarity such as dichloromethane [29,128,140,141,142,143,144,145,146,147,148,149,150], ethyl acetate [151,152], or chloroform [153,154]. In a few studies, liquid–liquid extraction was employed in order to extract SFN from various plant sources such as broccoli seeds after being defatted with light petroleum [155,156]. Then, hydrolysis of glucoraphane was performed in a solution mixture of dichloromethane: phosphate buffer containing phosphoric acid (H_3_PO_4_) (0.05 mol/mL) at pH 7.0 for 8 hr at 25 °C [155,157]. Afterwards, SFN extraction was performed by utilizing dichloromethane at room temperature and reconstituting the concentrated extracts in acetone prior to determination of the SFN content [155,156]. In some cases, it has been reported that a step prior to hydrolysis is added where glucoraphane is isolated and incubated with 2-(*N*-morpholino) ethanesulfonic instead of acidic water [148,158]. In a recent study, SFN isolation (from broccoli) was attempted by solid-phase extraction, with the aim to examine the different factors that can potentially affect the extraction process [139]. Initially, the separation capacity of three different cartridges (with different stationary phase), namely C18, silica, and amino, were evaluated, with the authors concluding that the ideal column was silica after elution with a range of solvents of different polarity (e.g., water, acetonitrile, dichloromethane, 0.1 M acetic acid, ethyl acetate, and hexane). This was attributed to the fact that SFN is a molecule of weak polarity and thus can easily be absorbed by a matrix of a similar polarity (e.g., silica) as opposed to C18 and amino cartridges [136]. Furthermore, the selection of a variety of different washing and elution solvents was evaluated indicating that ethyl acetate was the most appropriate one. This finding is in agreement with other studies, regarding SFN isolation from broccoli, using Solid Phase Extraction (SPE) [140,145,154,157,159,160] in combination with a solvent of medium polarity (e.g., dichloromethane) for achieving the highest rate of SFN recovery [139,161]. This finding comes into contradiction, with other studies claiming that the use of methanol as an elution solvent can lead to isolation of SFN with higher purity levels and recovery rates [162].

The last parameter to be investigated was the volume of the elution solvent. Various authors have suggested that increasing the elution volume of dichloromethane (from 2 to 6 mL) results in an increased amount of extracted SFN. However, in other studies, when the volume of dichloromethane exceeded 4 mL, the amount of extracted SFN remained almost constant [139,161]. Moreover, when 5 mL of methanol was utilized as an elution solvent, an SFN content of 94% purity was obtained [162]. Finally, the amount of elution solvents relies on many factors, including the quantity and viscosity of the analyte as well as the size of the cartridge.

### 3.2. Extraction of Iberin

The hydrolysis of glucoiberin yields IBN, which is in high abundance in the *Brassicaceae* and *Capparaceae* species and differs from SFN by one methylene unit in the aliphatic chain [163,164]. Despite its evidenced health benefits [165,166,167,168,169], very little is known about the extraction and purification of IBN, mainly due its chemical instability [170,171]. In a recent study, horseradish roots were used (as a source of IBN) as a raw material which was extracted overnight by maceration with ethyl acetate [34]. Afterwards, the non-soluble material was extracted with methanol, followed by ethyl acetate, and then washed with water. Finally, the highly lipophilic organic phase was sequentially chromatographed on various C18 cartridges [34]. Analysis of the fraction with ultra-violate (UV) absorbance at 242 nm, using high resolution electron spray ionization, demonstrated the presence of a single compound with a molecular formula of C_5_H_9_NOS_2_ and mass to charge ratio (m/z) equal to 164.0202 (in the positive mode) indicative of the successful extraction, isolation and purification of IBN [34]. In another study, an alternative way of IBN extraction was suggested, including an 8-hr shacking incubation of *Lesquerella fendleri* (L.) and *Physaria fendleri* seeds in dichloromethane (acidified with 0.1 M HCl) at room temperature followed by re-extraction of aqueous extracts with dichloromethane. Then, the dichloromethane extracts were sequentially washed with water and hexanes (in order to separate the hydrophobic and hydrophilic components), while total IBN content was determined with Gas Chromatography-Flame Ionization Detection (GC-FID) [85]. A post-incubation extraction procedure was previously suggested where a naturally-derived source of IBN was shaken-incubated, at room temperature, in de-ionized distilled water and then anhydrous magnesium sulfate and sodium chloride solutions were added [172]. Then, the mixture was extracted with dichloromethane and the resulting dichloromethane extracts were washed with hexane and water [172]. The yield of IBN being extracted by incubation with dichloromethane (acidified with 0.1 M HCl) was 90%, whereas the respective yield from the post-incubation extraction was only 48.6% [85,172]. The low yield of the post-incubation protocol can be credited to the instability of IBN in an aqueous environment [170].

Although IBN exerts structural similarities with SFN, it appears to be less stable in common solvents, thereby preventing its extraction using standard convectional extraction protocols [170,173,174]. For example, IBN is less stable in an aqueous environment rather than in methanol and ethanol. IBN’s instability, in alcohols, is due to its rearrangement into thiocarbamate or disubstituted thiourea when exposed to water or alcohol, respectively [53,170,173,175]. However, IBN remains relatively stable in acetonitrile. On the other hand, when raising the temperature of the column thermostat from 20 °C to 30 °C to 40 °C there appears to be a linear relation between temperature and instability of IBN. In addition, pH appears to be another crucial player that affects the stability of IBN as under alkaline conditions it can be decomposed, whereas an acidic environment considerably improves its stability [173,174,176,177]. Overall, given that the extraction and isolation of IBN is a trivial process (due to its instability), a possible approach to overcome this is by the substitution of water and alcohols with acetonitrile and the use of non-thermal techniques [170,171,178,179].

### 3.3. Extraction of Allyl Isothiocyanate

Among the various alkenyl ITCs, AITC (derived from the hydrolysis of sinigrin) is the most predominant one, characterized by its pungent odour and volatility. It is derived from cruciferous vegetables including *Brassica juncea* (L.) (Indian mustard), *Wasabia jamonica* (L.) *matsum* (wasabi), and *Armoracia rusticana* (horseradish) [180,181,182]. It has been shown to exert anticancer [183,184,185,186,187,188], anti-microbial [189,190,191], and anti-fungal activities [192,193,194,195], while also protecting against neurological disorders [196,197].

During the past decade, the extraction of AITC has been performed by either steam distillation or solvent extraction. In a study by Wu et al. (2009), the authors examined various solvents with different polarities such as cooking oil, petroleum, dichloromethane, diethyl ether, and ethyl acetate in an attempt to extract AITC from the hydrolysed dry roots of *Armoracia rusticana* [35]. The authors suggested that among these different extraction solvents, dichloromethane was the most effective one. In addition, a water extraction step was also added, according to which the dry (powdered) roots of *Armoracia rusticana* were mixed with acidified water (pH 4.0) for 24 hr at 20–40 °C. This step appears to maximize the extraction rate before final extraction with dichloromethane [35], a finding which is in agreement with the work of others [198,199,200,201]. Overall, hydrodistillation with dichloromethane is a more effective extraction method as AITC content is considerably higher than water extraction alone [35]. However, its major drawback is that all heat-liable volatile compounds (including AITC) can be decomposed and hydrolysed, thus leading to loss of sample quality [202,203]. Additionally, the utilization of heat (either for extraction purposes or for solvent evaporation) can induce loss of volatile compounds, thus minimizing the efficacy of the extraction process [202,203,204]. Finally, prolonged evaporation, under reduced pressure, can form traces of organic solvents in the final sample, thereby masking the abundance of the analyte [205].

In order to overcome loss of volatiles, an alternative extraction technology has been employed, called supercritical fluid extraction (SFE), according to which the extractant is separated directly from a mixture matrix using fluids above their critical values (supercritical fluids) [206,207,208,209]. SFE can be carried out at moderate temperatures, thus preventing thermal disintegration and loss of volatile compounds in addition to having no traces of organic solvents found in the final mixture [204,205]. Extraction of AITC was performed using supercritical CO_2_, with ethanol as a co-solvent, but because of the instability of ITCs in alcohols the study was discontinued [210]. Several attempts were made by other groups (e.g., by employing supercritical CO_2_ without any co-solvent or supercritical CO_2_ with either hexane, diethyl ether, ethyl acetate, and acetone) in order to minimize the instability of AITC. It was confirmed that the decomposition of AITC was prevented when hexane used as a co-solvent [35,37,201]. Li et al. (2010) identified moisture, temperature, and pressure of the gas as the critical factors to be considered for optimal SPE extraction of AITC [37]. Specifically, the authors reported that increasing the percentage of water improved the rate of AITC’s hydrolysis and hence its yield production and extraction [37]. However, excess of moisture can lead to degradation of the produced AITC into allyl allydithiocarbamate, which can further degrade into diallyl tetra- and penta-sulfide [37,199]. However, the excess of water on the SPE system can induce destruction on the porous morphology of the freeze-dried plant [37,202]. Therefore, it was concluded that the optimal moisture content of AITC (from wasabi), for SPE, should be maintained at 125% [37]. On the other hand, the same study has suggested that the temperature should be kept constant at 35 °C, since at this point the highest yield was noticed (408 mg AITC/100 g of wasabi roots) whereas at higher temperatures, 55 °C, the corresponding yield was lower (245 mg AITC/100 g of wasabi roots) [37]. This effect is likely due to the increase of AITC’s solubility as a result of the increased density of the supercritical CO_2_ [37]. Moreover, at lower temperatures no results were observed, probably due to the inactivation of myrosinase [37]. A similar effect was also reported in the case of the pressure of supercritical CO_2_ as the yield of extraction was higher under a pressure of 20 MPa and a temperature of 35 °C (104 mg of AITC/100 g of wasabi roots) when compared to a pressure of 25 MPa and a temperature of 35 °C (89 mg of AITC/100 g of wasabi roots) [37]. These findings are in agreement with those of other groups utilizing horseradish as the natural source of AITC (instead of wasabi) [211,212]. Eventually, Hu et al. (2009) claimed that “despite the fact that the SPE-CO_2_ extraction requires higher investment in equipment, is more efficient and less energy consuming and produces AITC of better quality” [35].

In an attempt to minimize the risk of AITC’s degradation, the extraction and isolation of sinigrin rather than AITC itself was utilized. In such studies, extraction with boiling water [48,209] or an aqueous–organic solvent system [213] was employed. To these ends, Powel et al. (2005) employed an optimized solid liquid protocol, taking into consideration the solvent composition, temperature, and pH [214]. In addition, Mohn et al. (2007) optimized critical parameters of pressurized liquid extraction, including solvent composition, particle size, temperature, and number of required extraction steps [215]. Furthermore, other research groups have focused on the extraction of sinigrin using ultrasonic-stimulated treatment [216]. This extraction technique relies on the disruption of cell wall promoting reduction in the particle-size and thus the mass transfer of the cell is enhanced due to the collapse of the cavitation bubble [217]. This technique is widely used in the separation and extraction of bioactive compounds, derived from natural sources, in the industrial scale [218,219,220]. Its major advantages involve improved extraction productivity, reduction of solvents, and overall protection of volatile and unstable phytochemicals [220,221,222]. In the case of sinigrin, it is recommended that the optimum condition for its maximal recovery is 57% ethanol, at 81 °C for 60 min, as under these conditions its yield increases by 70.67% when compared to other convectional extraction methodologies [216].

### 3.4. Extraction of Benzyl Isothiocyanate

BITC is the simplest form of benzylic ITC and it can be formed upon hydrolysis of its GSL precursor called Glucotropaeolin [223]. It is of high abundance in papaya seeds [39], garden [224], as well as Indian cress [225], and despite its simplicity in its chemical structure, its biological importance has been demonstrated in several studies [194,226,227,228,229,230,231,232]. Due to its numerous biological applications, its extraction has been challenging (like all ITCs) as it is thermally unstable and prone to degradation upon exposure to aqueous or alcohol environments [233,234,235]. In a study by Nakamura et al. (2007), the authors attempted to extract BITC from papaya seeds utilizing a liquid–liquid extraction protocol at room temperature [96]. The results of their methodology suggested that upon myrosinase inactivation the concentration of BITC was 213 μg/100 g of papaya seeds, substantially higher than during avoidance of the enzyme’s inactivation (68.7 mg/100 g of papaya seeds) [96]. Alternatively, solid–liquid extraction methodologies utilizing *Salvadora persica* (L.) as a source of BITC have been employed [236]. In such methodologies, the authors of various studies have avoided the use of hydroxylated solvents (alcohols) as BITC can form non-separable thiocarbamate conformers. Additionally, an increase in temperature also accelerates the formation of these thiocarbamate species [236,237]. For this purpose, three different classes of solvents were employed, namely chloroform (halogenated solvent), acetone (carbonyl contained solvent), and ethanol (hydroxylated solvent) [236]. The outcome of this study revealed that, among the three solvents, chloroform was the most effective as it gave the highest yield (2.1–2.4% *w*/*w*), suggesting that halogenated solvents are most efficient for BITC extraction [236]. These findings are in agreement with those of others, utilizing dichloromethane as an extraction analyte [237,238]. As expected, extraction of air dry or fresh plant material in ethanol or acetone leads to low recovery yields of 0.06–0.07% *w*/*w* or 1.12% *w*/*w* of BITC, respectively [236]. In the case of acetone, the authors suggested that the observed low recovery yield of BITC may be attributed to the water miscibility of acetone, which enhances the contact between BITC and polar components present in the plant [236,237,239]. Furthermore, hot continuous Soxhlet extraction with chloroform decreased the BITC content (0.88–0.92% *w*/*w*), possibly due to the volatile nature of BITC or its thermal degradation [202,240].

Even though there are numerous reports documenting that ITCs can be degraded under thermal conditions, a study by Nakamura et al. (2019) managed to extract BITC (of 80% purity) via heat distillation without the participation of any organic solvent [122]. Interestingly, in this study, there was no evidence of thiocarbamates formation (as a result of nucleophilic addition of water’s hydroxide in the ITC unit). In addition, the authors attempted to increase the stability of BITC, in an aqueous environment, through conjugation with glutathione (GSH), *N*-acetyl cysteine (NAC), or *L*-cysteine [122]. The results suggested that the decomposition of BITC is water was prevented by the addition of *L*-cysteine, but not with GSH nor NAC [122]. However, these findings contradict another study where authors examined the stability of benzylic-type ITCs in hydrodistillation conditions [241,242,243]. To this end, De Nicola et al. (2012) showed that upon exposure of BITC in an aqueous environment of greater than 90 °C, it is converted into benzylamine after 7 hr of treatment [241].

### 3.5. Extraction of Phenethyl Isothiocyanate

Watercress (*Nasturtium officinale*) is an enriched source of gluconasturtiin, the GSL precursor of PEITC [31]. It has been previously documented that PEITC is a powerful antioxidant [242,243,244,245,246,247,248,249,250,251], cancer chemo-preventive [252,253,254,255,256,257,258,259,260] and antimicrobial [261,262,263,264,265] agent. Several studies have demonstrated that the formation of PEITC is easily affected by various factors like temperature and pH. In addition, the cooking process of watercress itself inactivates myrosinase, which is susceptible to thermal denaturation, hence preventing the conversion of gluconasturtiin into PEITC [87,266].

The extraction of PEITC utilizing a simple ultrasound extraction methodology has been proposed by Fusari et al. (2019), according to which defatted rocked seeds were sonicated at 40 kHz, 600 W for 5 min in the presence of 0.1 M bicarbonate buffer (pH 8.1). The formed solution was stirred at 37 °C for 2 hrs to ensure complete conversion of GSLs into their respective ITCs. Finally, the hydrolysis solution was then extracted (from acetonitrile/dichloromethane) prior to High Performance Liquid Chromatography couple to a diode array detector (HPLC-DAD) analysis and separation [267]. A similar liquid–liquid extraction methodology using safflower oil was used for the extraction of non-polar ITCs, including PEITC, from natural sources [268]. More specifically, the authors homogenized pulverized powder of watercress, in deionized water, to allow hydrolysis of GSLs. To this solution, safflower oil (containing antioxidants like tocopherol and carotenoids) was added in order to extract PEITC from the aqueous hydrolytic mixture. Interestingly, the safflower oil and the antioxidants did not interfere with PEITC, and they protected the analyte from its oxidation and degradation [268]. Additionally, the hydrophobic nature of the oil allows the maximum extraction of PEITC without the need for further re-extraction (as it usually happened during standard liquid–liquid extraction) [268]. In another conventional solvent extraction model, utilized by Rodrigez et al. (2016), hydrolysis of gluconasturtiin was performed by incubating freeze-dried watercress humidified with 125% of distilled water, at 35 °C for 60 min, under atmospheric pressure [269]. The aqueous hydrolysis mixture, containing PEITC, was then extracted with hexane pre-warmed at 40 °C [269]. The isolated extracts contained 20.5 ± 2.7 μmol of PEITC/g of freeze-dried watercress as indicated by GC-MS coupled to UV [269]. The same extraction methodology was utilized by Ji et al. (2003) to determine the total PEITC content in human plasma and urine samples [270].

The use of SFE is widely applied for the extraction of bioactive phytochemicals from natural sources, including the recovery of PEITC from watercress. Initially, the raw freeze-dried material was incubated at 25–35 °C, under 25 MPa pressure, for 30–120 min to ensure the hydrolysis of the GSL precursors. The extraction of ITCs was achieved via supercritical CO_2_ in the presence or absence of ethanol at 35 °C and 25 MPa [269]. Overall, when supercritical CO_2_ extraction was utilized in the absence of any solvent, selectively isolated PEITC of high purity and content (31.7 ± 1.6 μmol PEITC/g of freeze-dried material) was noted. On the contrary, when a CO_2_: ethanol mixture was used, the concentration of the recovered PEITC decreased to 17.2 ± 2.7 μmol PEITC/g of freeze-dried material [269]. The difference in the recovered PEITC content can be attributed to the fact that ITCs are generally unstable under conditions including hydroxylated solvents (e.g., ethanol) [269]. Overall, extraction with supercritical CO_2_ (with or without ethanol) is a considerably more effective extraction methodology for PEITC (from a watercress source) as it has the capacity for recover higher quantities and purity levels. However, the expensive and specific instrumentation required for the implementation of such methodologies constitutes a major disadvantage.

Alternatively, Coscueta et al. (2020) exploited, for the first time, the extractive capacity of aqueous micellar systems composed of two non-ionic surfactants for the extraction of PEITC from watercress in an attempt to employ economic alternatives to flammable, toxic, and expensive organic solvents [98]. Non-ionic surfactants are amphiphiles which have been previously utilized for the extraction and purification of bioactive compounds [271,272,273,274]. Examples of non-ionic surfactants are Triton (X-100 and X-114), Brij (−30, −56 and −97), Genapol (X-080), and to a lesser extent, the Tergitols (15-S-X). A major advantage of using micelles is that their transparency in the 240–280 nm region allows the monitoring of aromatic or conjugated systems more easily. In addition, non-ionic surfactants have the capacity for developing interactions with wither hydrophobic or hydrophilic parts of the different molecules, allowing their extraction and purification more effectively [98,275,276,277,278]. Specifically, upon extraction of PEITC (from watercress) with either alcohol ethoxylate non-ionic surfactants (e.g., Genapol X-080 and Tergitol 15-S-7) or a range of organic solvents of decreasing polarity (e.g., *n*-hexanes and a mixture of acetonitrile/chloroform; 10:7), results suggested that its extraction with non-ionic surfactants was the most efficient one. The PEITC content obtained from the extraction with Tergitol 15-S-7 and acetonitrile/chloroform did not differ significantly from n-hexane, whereas in the case of Genapol X-080, the respective PEITC content was decreased [98]. Overall, it was suggested that the optimum conditions for PEITC should be 2.0% m/m of non-ionic surfactant at 25 °C and a pH range from neutral to slightly basic, conditions at which the concentration of the analyte was found to be 2887 and 2971 µg of PEITC/g of freeze-dried watercress with Genapol X-080 and Tergitol 15-S-7, respectively [98]. Finally, Table 4 summarizes the various methodologies used in the extraction of various ITCs from their natural sources.

## 4. Quantification of the Extracted ITCs Content

**Figure 5:**
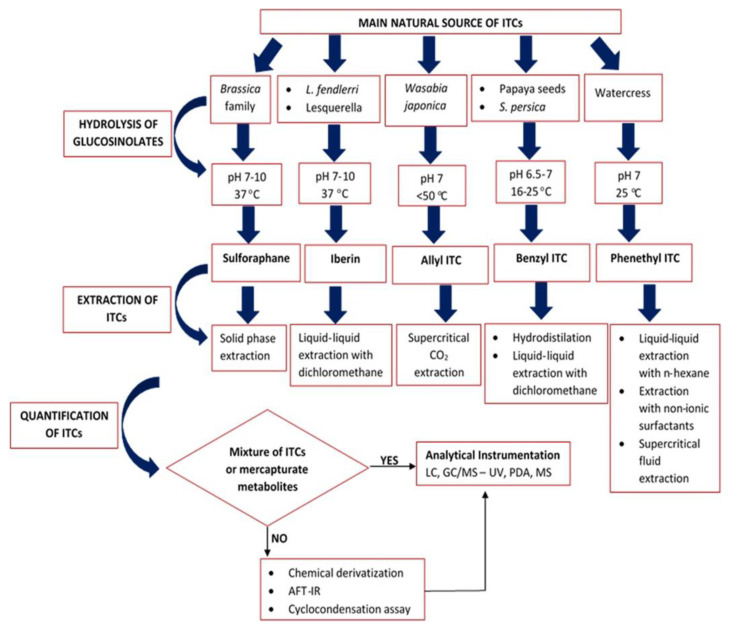
Graphical presentation of the proposed methodological pipelines for the determination of ITCs from various natural sources, taking into consideration the efficacy of each approach based on recovery yield of isolated ITCs.

The isolation and quantification of ITCs from various natural sources can be a challenging process. This is mainly because each plant produces different ITCs and their extraction procedure varies depending on their physicochemical nature (polarity, stability). Furthermore, it appears that the hydrolysis of GSLs can also be a highly demanding procedure as alterations in the hydrolytic conditions (temperature and pH) can induce denaturation and inactivation of myrosinase, hence limited ITCs production. Additionally, such effect on myrosinase can promote the chemical rearrangement of GSLs into nitriles, thus posing a toxic profile. Another characteristic factor affecting the quantification of ITCs is their volatility, which makes them vulnerable to degradation under thermal conditions. As a result, several procedures have been utilised for the extraction of specific ITCs. Overall, multiple protocols have been employed, allowing the quantification of ITCs either as a mixture or the major extraction product. These methodological pipelines are summarized in Figure 5.

### 4.1. Chemistry and Reactivity of ITCs

The chemistry of ITCs relies mainly on the electrophilicity of their -N=C=S moiety, allowing them to react with several nucleophilic centres including oxygen, nitrogen, and sulfur, thereby forming dithiocarbamates, thioureas, and dithiocarbamates, respectively [279,280]. An example is the Edman degradation during which PEITC reacts with the amino group of amino acids, under alkaline conditions, forming an intermediate which is then rearranged into substituted phenethylthiohydantoin products [281,282]. Furthermore, the reactivity of ITCs is also facilitating their metabolism. To this end, ITCs absorbed in the liver then enter the mercapturic acid pathway in which the carbon atom of the –N=C=S moiety reacts primarily with the cysteine thiol of GSH in a reaction catalyzed by glutathione *S*-transferase (GST) [283]. The resulting conjugate (e.g., glutathione dithiocarbamate) undergoes further enzymatic modifications yielding sequentially a cysteinyl glycine (in a reaction facilitated by γ- glutamyl transpeptidase) and *N*-acetyl cysteine (NAC) (by a *N*-acetyl transferase) conjugates, which are excreted in urine [284,285,286,287].

### 4.2. Cyclo-Condensation Assay

**Figure 6:**
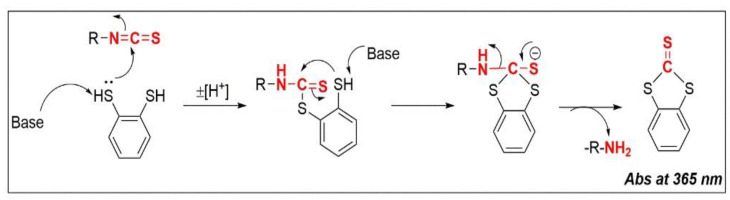
The cyclo-condensation assay is used for the quantification of ITCs. Briefly, it involves the nucleophilic attack of both vicinal thiols of 1,2-benzenedithiol into the electrophilic carbon atom of ITC, leading to the formation of a stable 1,3-benzodithiole-2-thione product which absorbs at 365 nm.

A study by Fabian et al. (1967) was the first one that attempted to quantify ITCs [288]. This was achieved by reacting propyl ITC with 2,3-dimercaptopropanol in aqueous solution of moderated basicity [288]. The continuous ultra-violet (UV) monitoring of the reaction suggested the (rapid) formation of a product carrying the dithiocarbamate moiety with characteristic maximal absorption at 270 nm. Further incubation of this dithiocarbamate intermediate led to the development of a high intensity spectra with a single isosbestic point at 287 nm [288]. Later, in a study performed by Zhang et al. (1992), the authors isolated, purified, and characterized the isolated product, documenting the presence of a cyclic thione called 4-hydroxymethyl-1,3-dithiolane-2-thione [289]. The mechanism of the reaction suggested that the centre carbon atom of the isothiocyanate group of ITCs undergoes nucleophilic attack by a thiol group of a vicinal dithiol system, leading initially in the formation of the unstable dithiocarbamate molecule [290]. Intramolecular cyclization and consequent degradation of dithiocarbamate into a five membered 1,3-dithiolane-2-thione was shown to be promoted by a second nucleophilic attack of the unreacted thiol group (adjacent thiol group), upon prolonged standing [290]. Despite the fact that intramolecular cyclization between ITCs and vicinal amino or thiol groups had previously been demonstrated, a cyclo-condensation with vicinal dithiols had not been reported before thereby suggesting a new chemical reaction of ITCs [290,291]. This cyclo-condensation reaction was subsequently shown to be useful in generating primary amines from alkyl and aryl ITCs, but it was also realized that this reaction can be applied into the quantification of ITCs by measuring the absorbance of the formed cyclic product [291,292]. Further optimization of the cyclo-condensation reaction was performed by screening several vicinal dithiol substrates, demonstrating that reaction of ITCs with 1,2-benzenedithiol (BDT) can lead to the rapid formation of a very stable 1,3-benzodithiole-2-thione with high molar extinction coefficient at the long wavelength (ε = 23,000 M^−1^ cm^−1^ at 365 nm) (Figure 6) [289].

The cyclo-condensation reaction can be performed by all aliphatic and aromatic ITCs, except for the tertiary ones (e.g., *tert*-butyl ITC) [289]. Several attempts have been made to promote the reaction, such as increasing the concentration of BDT to 50 mM (rather than 4 mM) or by introducing prolong incubation periods (15 hr at 65 °C) [289]. Despite several optimization steps, only 20% of *tert*-butyl ITC was shown to be able to react. The low reactivity of the tertiary ITCs can be attributed to a potential steric hindrance as the tertiary carbon atom which can block the side where thiol can react to the central carbon of -N=C=S. In addition, the electronegativity of the adjacent nitrogen might be reduced due to the presence of the three methyl groups (in the case of *tert*-butyl ITC), preventing the elimination of amine [289]. The quantification of ITCs from blood, urine, plant extracts, etc., via the cyclo-condensation assay, does not require their prior modification. In the past, labelling of the ITCs with radioactive ^14^C or deuterium was essential for their quantification [267,285,293,294,295,296]. With respect to the sensitivity of the assay, it has been previously reported that the limit of cyclic adduct detection can be as low as 10 pmol, according to simple reverse phase HPLC area integration, using a C18 column and isocratic mobile phase consisting of 80% methanol, 20% water, and a photodiode array detection unit [297]. It has also been suggested that the sensitivity could be lowered at least another 10-fold by utilizing a solid phase extraction of the ITC coupled 1,3-benzodithiole-2-thione via a reverse phase cartridge (Sep-Pak) [298]. Since the cyclisation reaction occurs under relatively basic conditions, the substitution of phosphate buffer with a borate one (500 mM at pH 9.25) can increase the stability and the sensitivity of the detection of ITCs in urine samples. Alternatively, the use of aprotic polar solvents (e.g., acetone, acetonitrile, dimethylsulfoxide, and dimethylformamide) can facilitate the solubility of the reaction solution in high protein-containing samples [298].

Finally, the main disadvantage of the cyclo-condensation assay is that it does not allow the discrimination of each individual ITC or dithiocarbamate molecule in a mixture, since both molecules form the same cyclic product. As a result, the urinary levels of ITC equivalents (including ITCs and dithiocarbamates) were inversely associated with carcinogen-induced DNA damage as well as for the risk of developing stomach, lung, colon, and breast carcinomas [267,296,297]. Furthermore, the chance of having a positive false during the detection is likely, as a number of chemical entities can react with BDT in the same way as ITCs do [287,294].

### 4.3. Quantification via ITCs Chemical Derivatization

In many cases, the quantification of ITCs appears to be a challenging procedure due to their limited stability in either aqueous or methanolic media solutions [170,299,300,301]. As a result, in many studies, the quantification of ITCs was performed either by measuring the concentration of the degradation products or by derivatizing the ITC extracts prior to quantification. The chemical derivatization of ITCs results in the formation of considerably more stable compounds, allowing their quantification by analytical instrumentation.

In the case of PEITC, a study by Negrusz et al. (1998) used the degradation product phenethylamine as a marker to quantify the content of PEITC found in dog’s plasma by using gas chromatography coupled to mass spectrometry with chemical ionization [302]. In another study, the determination and quantification of PEITC in human plasma and urine was performed by derivatizing these samples with ammonia [270,303]. Chemical derivatization of samples is an approach that results from the major disadvantage of the cyclo-condensation assay which does not allow the distinction between PEITC from other species like phenethyl-*N*-acetyl cysteine, dithiocarbamates, etc. [270,303]. In addition, the derivatization allows the recovery of higher amounts of ITC as well since there is no loss, due to volatility, during the evaporation process [270,303]. Even though mercapturate metabolites (e.g., phenethyl-ITC-*N*-acetyl cysteine) can also be converted to phenethyl thiourea, after treatment with ammonia, these can be removed during the hexane-based extraction phase [270]. Therefore, any false positives when determining PEITC content are potentially prevented. For instance, unlike the cyclo-condensation reaction, the resulting thiourea product is unique for each individual ITC and so interference from any other ITCs (which might present in the sample) is prevented [270,303]. The same approach was followed by another study when determining the content of PEITC in human blood samples [303,304].

Finally, others have postulated that the accurate quantification of ITCs (via reverse-phase Liquid Chromatography on C18 cartridges) of low polarity (e.g., hepty-ITC or SFN) is prevented due to their aqueous precipitation. To overcome this effect, the authors of another study derivatized the extracted ITCs with mercaptoethanol, thus allowing the formation of more polar and consequently more water-soluble compounds [305]. This derivatization facilitated the analysis of their corresponding ITCs using an aqueous or water-containing mobile phase, in addition to increasing the overall sensitivity of the assay’s detection and quantification limits [305].

### 4.4. Quantification via Analytical Instrumentation

#### 4.4.1. Attenuated Total Reflectance Infrared Fourier Transform (ATR-FT-IR) Spectroscopy

As it is mentioned above, the instability of ITCs, as well as their precipitation in aqueous mobile phases, led many researchers to derivatize ITCs in an attempt to minimize these factors. However, the derivatization of ITCs introduces extra steps into the experimental methodology of quantification, with major impacts including cost effective reagents as well as the possibility of minimizing the yield of product formation. Therefore, Revelou et al. (2017), for the first time, utilized attenuated total reflectance infrared Fourier transform (ATR-FT-IR) spectroscopy and partial least-squares for the determination of total isothiocyanate content in broccoli [306]. For this purpose, each spectrum was recorded using a zinc–selenide (ZnSe) 45 flat plate against a ZnSe background and the spectra smoothing was performed by applying a Savitsky–Golay algorithm [306]. The increment in the concentration of the analyte standard was accompanied with an increment in the absorption at spectral region; 2150–2020 cm^−1^, whereas the doublet peak at 2120 and 2058 cm^−1^ of the same spectra has been correlated with asymmetric stretching of N=C=S functionality, since blank samples did not detect such signals [307]. Analysis of the broccoli extracts suggests the appearance of the characteristic isothiocyanate bond stretching (by means of doublet peak) verified the above findings. Those findings proposed the utilization of ATR-FTIR methodology as an alternative quick, reproducible, and accurate approach for the determination and quantification of total isothiocyanate content in *Brassica* vegetables [306]. Despite the encouraging results that were obtained, the authors did not characterize this technique in terms of selectivity. Since the quantification of ITCs via ATR-FTIR relies on the signal’s intensity, it should be expected, like cyclo-condensation, that the same absorbance would be for all ITCs. Therefore, this method can only be used and applied in cases where only one ITC is omitted.

#### 4.4.2. Chromatographic Approaches

The use of analytical instrumentation (e.g., liquid (LC) and/or gas chromatography (GC)) coupled to either a UV or PDA (photodiode detector array) detection unit or tandem mass spectrometry (MS/MS) has been widely used in the isolation and quantification of phytochemicals, including ITCs. Initially, the quantification of ITCs was performed on gas chromatography coupled to mass spectrometry. Marton et al. (2013) quantified the total ITC content (from mustard seeds) by employing a GC-MS/MS-based approach, according to which each analyte was eluted through a preheated column and then ionized via electron ionization [38]. The authors concluded that this methodology was more time-cost efficient, simple, reproducible, and sensitive [38]. In another study, the quantification of SFN from broccoli was attempted by using GC-MS instrumentation [308]. In this case, the authors stated that this methodology caused approximately 80% degradation of SFN into 3-butenyl ITC [308]. To minimize the thermal degradation (down to 5%), the authors utilized a combination of a 1.5 mm direct inlet liner in conjugation with fast initial injection flow. These conditions improved the on-column injection, thermal degradation, and also prolonged the column deterioration. These findings are in agreement with a study by Jin et al. (1999) which confirmed thermal degradation of SFN at 50 °C and 100 °C (temperatures that GC instrumentation operates) [309]. To this end, an alternative way of quantifying the content of ITCs (in Chinese herbs) was proposed, during which a cyclo-condensation assay was utilized, followed by the monitoring of the quantity of the resulting 1,3-benzodithiole-2-thione molecule by GC-MS and selective ion recording, to increase the sensitivity of detection [310]. The authors concluded that the GC-MS method for the analysis of ITC content in vegetables and herbs is an optimum one since 1,3-benzodithiole-2-thione is highly stable, thus allowing for robust data of high sensitivity [310]. Eventually, numerous reports suggested the use of GC-XX as an optimal instrumentation for the determination and quantification of ITC content in vegetables [302,311,312,313,314]. However, the thermal decomposition of ITCs during the analysis is an important factor that should be taken into consideration during quantification.

In many studies, the quantification of extracted ITCs has been performed by utilizing HPLC instrumentation coupled to several detection sources (e.g., MS, PDA, or UV). Zheng et al. (2014) attempted to quantify the PEITC content in human plasma, taking into consideration all previously published methodologies together with all the associated limitations (e.g., duration, scale, sensitivity, and selectivity) [315]. For the first time, the authors utilised atmospheric pressure chemical ionization (APCI) in order to detect and quantify PEITC. The authors recommended the use of this ionization mode to be considerably more effective for the ionisation of PEITC when compared to the electrospray ionisation using the LC-MS/MS platform [315]. This is because the protonation of phenethyl ITC under electron spray ionisation (ESI) occurs in the liquid phase, whereas the respective protonation under APCI occurs in the gaseous phase. Furthermore, when considering the instability of PEITC, simple protein precipitation (with acetonitrile) was utilized prior to exposure of the sample at low temperature under acidic conditions. The quantitation was then performed by using multiple reaction monitoring (MRM) mode for the transition: PEITC, [M + H]^+^ m/z 164.0→m/z 130.0 [312]. Eventually, the authors concluded that this methodology comprised a simple, sensitive, and valid approach for the quantification of PEITC in human plasma samples. A later study by Yu et al. (2020) suggested a less expensive but yet effective methodology for the determination of SFN content by utilizing a UHPLC-MS/MS-based approach [316]. The overall analysis relied on the MRM mode, according to which the SFN content was quantified using the following transition [M+H]^+^ m/z 178.3 →m/z 114.2 [316]. Similar approaches were employed by others in an attempt to quantify SFN content from various natural sources [30,162,317,318].

## 5. Conclusions

This review summarises the experimental pipeline followed in order to extract and quantify major ITCs from naturally-derived sources. The entire process (which varies across the different classes of ITCs) involves the conversion of GSLs into ITCs prior to their extraction. The major limitation(s) of this pipeline is that there is not an established universal extraction and quantification protocol which can be applied to all ITCs. Therefore, their quantitative extraction (from a single natural source) can be a challenging process. In addition, the chemical instability of the ITCs moiety makes them prone to destabilisation when exposed to various solvents and/or high temperature. Finally, several challenges are observed during the entire process of isolation and quantification of ITCs, even if the same protocol is followed. To an extent, this can be potentially attributed to the different cultivation and growing conditions of the primary natural sources.

Given the crucial role of ITCs in modulating several cellular cascades, it is of utmost importance to be able to determine their content in various naturally-derived sources. In future studies, the development of a single universal pipeline should be a priority in order to allow the quantitative extraction of ITCs from naturally occurring sources. This, in turn, would potentially allow further exploitation of ITCs in drug development, among other applications.

## Figures and Tables

**Table 1:** Natural sources of ITCs including genus species.

ITCs Source	Genus Species (Sub Species)	Isothiocyanates (ITCs)	Ref
Broccoli	*Brassica oleracea* var*. italica*	sulforaphane (SFN)	[28,29]
Curly Kale	*Brassica oleracea* var. *laciniata* (L.)	SFN	[30]
Cauliflower	*Brassica oleracea* var. *cauliflora*	SFNphenethyl isothiocyanate (PEITC)	[30,31]
Cabbage	*Brassica rapa* var*. pekinensis*	SFN	[32]
Brussel sprout	*Brassica oleracea* var. *gemmifera*	SFN	[33]
Horseradish	*Armoracia lapathifolia* (L.) Gilib	iberin (IBN) allyl isothiocyanate (AITC)	[34,35]
Radish	*Raphanus sativus* (L.)	IBN	[34]
Watercress	*Nasturtium officinale*	PEITC	[36]
Wasabi roots	*Eutrema japonicum* (L.) Koidz.	AITC	[37]
Mustard seeds	*Sinaptis alba*	benzyl isothiocyanate (BITC)	[38]
Papaya seeds	*Carica* papaya (L.)	BITC	[39]

**Table 2:** Optimum pH conditions for complete conversion of various GSLs into their respective ITCs in various plants.

Plant Species	pH	Glucosinolate (GSL)	Isothiocyanate (ITC)	Extraction Yield (%)	Refs
*Eruca sativa* var. *sativa* *Erysimum allionii*	10.0	glucoerucin	erucin	98.7 96.6	[61,85,86,87,88,89,90,91,92,93,94,95,96,97,98,99,100,101,102]
*Brassica hirta* (L.) Moench	7.0, 10.0	4-hydroxybenzyl	4-hydroxybenzyl	29.6	[103]
*Lesquerella fendleri*	7.0, 10.0	glucoiberin	IBN	90.0	[85]
*Brassica* vegetables *Mattiola longipetala*	7.0, 10.0	glucoraphin	SFN	53.0 15.0	[61,91,92,93,94,95,96,97,98,99,100,101,102,103,104]
*Lobularia maritima* (L.) Desv *Capsella bursa-pastoris* (L.) Medik	7.0, 10.0	3-butenyl	3-butenyl	~100.0	[85]
*Erysimum cheiri* (L.) Crantz	7.0, 10.0	glucocheirolin	cheirolin	98.3	[85]
*Lobularia maritima* (L.) Desv	7.0, 10.0	lesquerellin	lesquerellin	96.3	[85]
*Nasturtium officinale*	7.0	gluconasturtiin	PEITC	89.095.0	[87,105,106]
*Carica* papaya (L.) *Tropaeolum majus* (L.) *tropaeolaceae* *Lepidium sativum* (L.)	6.5–7.0	glucotropaeolin	BITC	n.d.	[107]
*Brassica rapa* (L.) *oleifera*	8.0	3-butenyl glucosinolate	3-butenyl ITC	40.0	[56]
Arabidopsis thaliana (L.) Heynh	6.5	2-propenyl glucosinolate	2-propenyl ITC	32.0	[56]
*Brassica oleracra* var. *italica*	5.5	4-(methylsulphinyl) butyl glucosinolate	4-(methylsulphinyl) butyl isothiocyanate	82.0	[56]
*Armoracia rusticana*	7.0	sinigrin	AITC	61.0	[108]

**Table 3:** Optimum temperature(s) for complete degradation of GSLs into their respective ITCs in various plants.

Plant Species	Temperature	Glucosinolate (GSL)	Isothiocyanate(ITC)	Extraction Yield (%)	Refs
*Brassica* vegetables	37 °C	glucoraphane	SFN	53.0	[91]
*Nasturtium officinale*	25 °C	gluconasturtiin	PEITC	89.0	[87] [106]
*Brassica rapa* var. *oleifera*	25 °C	3-butenyl glucosinolate	3-butenyl ITC	40.0	[55]
*Arabidopsis thaliana* (L.) Heynh	37 °C	2-propenyl glucosinolate	2-propenyl ITC	32.0	[55]
		3-butenyl glucosinolate 2- buteny l glucosinolate	3-Butenyl ITC 2-Butenyl ITC	n.d. n.d.	[62]
*Brassica oleracra* var. *italica*	37 °C	4-(methylsulphinyl)butyl glucosinolate	4-(methylsulphinyl)butyl isothiocyanate	82.0	[56]
*Carica* papaya (L.)	16–18 °C	glucotropaeolin	BEITC	85.6	[119]
*Salix alba* (L.) *maire*	<50 °C	sinigrin	AITC	n.d.	[112]
*Brassica nigra* (L.) *nigra*	<60 °C	sinigrin	AITC	n.d.	[112]
*Brassica juncea* (L.) Czern.	<50 °C	sinigrin	AITC	n.d.	[112]

**Table 4:** Optimum conditions for the extraction of ITCs from naturally-derived sources.

Plant Source	Isothiocyanates (ITCs)	Extraction Methodology	Extraction Yield (%)	Refs
Broccoli	SFN	Solid phase extraction	94	[139]
*Matthiola* *longipetala*		Liquid-liquid extraction (with n-*hexane*)	31.9	[85]
*Lesquerella fendleri* (L.)	IBN	Liquid-liquid extraction (with dichloromethane)	48.6	[85]
*Physaria fendleri*			56.7	[210]
*Wasabia Japonica* (L.) *matsum*	AITC	Suprecritical fluid (CO_2_) extraction	79.1	[35]
*Armoracia rusticana*		Hydrodistillation	61	[35]
		Liquid-liquid extraction (with diethyl ether)	96.5	[35]
Green papaya	BITC	Hydrodistillation	80	[122]
*Salvadora persica* (L.)		Liquid-liquid extraction (with dichloromethane)	75	[236]
		Liquid-liquid extraction (with chloroform)	40	[237]
*Nasturdium officinale*	PEITC	Extraction with non-anionic surfactants	94	[98]
		Liquid-liquid extraction (with *n*-hexane)	98.7	[106]
		Suprecritical fluid (CO_2_) extraction	87.4, ND	[206,271]
*Lobularia maritima*	6-methylthiohexyl isothiocyanate	Liquid-liquid extraction (with *n*-hexane)	96.3	[85]
*Matthiola* *longipetala*	erysolin	Liquid-liquid extraction (with *n*-hexane)	38.7	[85]
	cherolin		18.7	

## Abbreviations

AITC	allyl isothiocyanate
APCI	atmospheric pressure chemical ionization
ATR-FT-IR	attenuated total reflectance infrared Fourier transform spectroscopy
BDT	1,2-benzenedithiol
BITC	benzyl isothiocyanate
CDK	cyclic depended kinase
DAD	diode-array detection
EDTA	ethylenediaminetetraacetic acid
ESI	electron spray ionisation
ESP	epithiospecifier protein-like factors
FID	flame ionization detection
GC	gas chromatography
GLs	glucosinolates
GSH	glutathione
GST	glutathione *S*-transferase
HPLC	high performance liquid chromatography
IBN	iberin
ITC	isothiocyanates
MRM	multiple reaction monitoring
MS	mass spectrometry
MS/MS	tandem mass spectrometry
m/z	mass to charge ratio
NAC	*N*-acetyl cysteine
PDA	photodiode-array detection
PEITC	phenethyl isothiocyanate
ROS	reactive oxygen species
SFE	supercritical fluid extraction
SFN	sulforaphane
SPE	solid phase extraction
UV	ultra-violate
